# Kinetic Modeling of In Vivo K^+^ Distribution and Fluxes with Stable K^+^ Isotopes: Effects of Dietary K^+^ Restriction

**DOI:** 10.3390/ijms25179664

**Published:** 2024-09-06

**Authors:** Jang H. Youn, Stefania Gili, Youngtaek Oh, Alicia A. McDonough, John Higgins

**Affiliations:** 1 Department of Physiology and Neuroscience, University of Southern California Keck School of Medicine, Los Angeles, CA 90089, USA; youngoh@usc.edu (Y.O.); mcdonoug@usc.edu (A.A.M.); 2 Department of Geosciences, Princeton University, Princeton, NJ 08544, USA; tefigili@gmail.com (S.G.); jahiggin@princeton.edu (J.H.)

**Keywords:** potassium homeostasis, renal excretion, skeletal muscle, potassium uptake, isotope ratio analysis

## Abstract

Maintaining extracellular potassium (K^+^) within narrow limits, critical for membrane potential and excitability, is accomplished through the internal redistribution of K^+^ between extracellular fluid (ECF) and intracellular fluid (ICF) in concert with the regulation of renal K^+^ output to balance K^+^ intake. Here we present evidence from high-precision analyses of stable K^+^ isotopes in rats maintained on a control diet that the tissues and organs involved in the internal redistribution of K^+^ differ in their speed of K^+^ exchange with ECF and can be grouped into those that exchange K^+^ with ECF either rapidly or more slowly (“fast” and “slow” pools). After 10 days of K^+^ restriction, a compartmental analysis indicates that the sizes of the ICF K^+^ pools decreased but that this decrease in ICF K^+^ pools was not homogeneous, rather occurring only in the slow pool (15% decrease, *p* < 0.01), representing skeletal muscles, not in the fast pool. Furthermore, we find that the dietary K^+^ restriction is associated with a decline in the rate constants for K^+^ effluxes from both the “fast” and “slow” ICF pools (*p* < 0.05 for both). These results suggest that changes in unidentified transport pathways responsible for K^+^ efflux from ICF to ECF play an important role in buffering the internal redistribution of K^+^ between ICF and ECF during K^+^ restriction. Thus, the present study introduces novel stable isotope approaches to separately characterize heterogenous ICF K^+^ pools in vivo and assess K^+^ uptake by individual tissues, methods that provide key new tools to elucidate K^+^ homeostatic mechanisms in vivo.

## 1. Introduction

K^+^ homeostasis is critically important to health because it determines the membrane potential, and thus excitability and contractility, in cardiac and skeletal muscles and nervous tissues. Extracellular fluid (ECF) K^+^ homeostasis is achieved by the concerted actions of multiple organs and tissues [1,2,3]. K^+^ enters the body with the diet and is absorbed in the gut into the blood. Despite wide variations in K^+^ intake, plasma or ECF K^+^ concentration ([K^+^]) remains relatively constant thanks to the kidneys, which have a remarkable capacity to regulate K^+^ excretion (output) to match K^+^ intake (input) and play a major role in chronic K^+^ balance [4,5,6]. In addition, skeletal muscles, the major K^+^ reservoirs in the body, provide a buffering capacity to K^+^ in the ECF by shifting K^+^ between the ECF and intracellular fluid (ICF) [7,8]. For example, the K^+^ content of a typical meal would result in a dangerous rise in ECF [K^+^] were it not for the effects of insulin (a hormone secreted by the pancreas after a meal) that stimulates the net movement of K^+^ from ECF to ICF, mainly in skeletal muscles. In addition, the gut sensing of dietary K^+^ appears to generate signals for regulating renal K^+^ excretion and extrarenal K^+^ shift [9,10,11]. Thus, ECF K^+^ homeostasis is maintained by a concerted effort of the gut, kidneys, pancreas, and muscles (and probably the brain [12]), and crosstalk exists among these organs/tissues for ECF K^+^ homeostasis.

Despite the marked progress made in understanding ECF K^+^ homeostasis, many important issues remain unaddressed. For example, it is often difficult to assess the relative contributions of individual tissues (renal vs. extrarenal; skeletal muscle vs. other extrarenal tissues) to ECF K^+^ regulation [13]. Understanding the roles and interactions of multiple organs and tissues in K^+^ homeostasis would require a method that can estimate K^+^ distribution and fluxes in vivo. Previously, we introduced a novel approach of quantifying K^+^ distribution and fluxes in vivo using stable K^+^ isotopes [14]. ^41^K and ^39^K are stable isotopes of K^+^ with natural abundances of roughly 6.7% and 93.3%, respectively. Using an approach similar to radioisotope labeling with ^42^K [15,16], we demonstrated that K^+^ enriched in the less abundant isotope (^41^K) can be administered in rats to alter plasma ^41^K/^39^K ratios and explored quantitative changes in K^+^ fluxes associated with both K^+^ excretion and redistribution among internal K^+^ pools. Although a model with two internal K^+^ pools (ECF and ICF; 2-compartment [2-C] model) was able to fit the data, the time course of ^41^K/^39^K ratios predicted for the ICF pool was not well matched to that observed in red blood cells (RBCs), measured as an ICF pool, indicating a limitation of this simple model assuming a single homogenous ICF K^+^ pool.

Here, we present a modified protocol (i.e., experimental duration and sampling number) that allows for the identification of two distinct ICF pools, one that exchanges K^+^ rapidly with the ECF (“fast” K^+^ pool) and one that exchanges K^+^ with the ECF more slowly (“slow” K^+^ pool). We conducted experiments in rats with increased duration of observation (from 3 to 5 h) and number of samplings (from 10 to 18–20) and tested if this extended protocol allows us to identify a three-compartment (3-C) model. The results indicate that the 3-C model could be robustly identified from the experimental data. In addition, 3-C modeling detected significant effects of dietary K^+^ restriction on K^+^ distribution and fluxes, disclosing selective effects of K^+^ restriction to decrease the slow vs. fast ICF K^+^ pool size and specific effects to decrease K^+^ efflux activities observed in both the fast and slow K^+^ pools. These results provide novel insights into the regulation of ECF K^+^ homeostasis with K^+^ deficiency and a foundation for future studies exploring K^+^ homeostasis under different physiological states.

## 2. Results

### 2.1. Effects of ^41^K Infusion on Plasma ^41^K/^39^K Ratios

**Figure 1:**
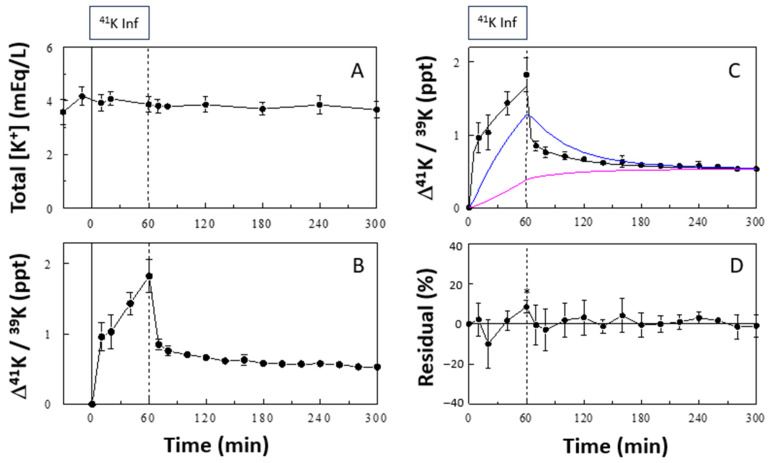
Effects of ^41^K infusion on plasma [K^+^] (**A**) and Δ^41^K/^39^K (**B**). In (**C**), a 3-C model fit to the plasma profile of Δ^41^K/^39^K is shown together with the predicted profiles of Δ^41^K/^39^K in the fast (blue curve) and slow (pink curve) ICF pools (ICF2 and ICF3, respectively). In (**D**), residuals of the model fit (differences between observed and model-estimated values), expressed as % of observed values, are shown. Boxes indicate the ^41^K infusion period. Isotope ratios are expressed as increments (Δ) from basal values. Residuals were statistically different from 0 only at 60 min (2-tailed t test; *p* values were adjusted for multiple comparisons by the Bonferroni method). Data are means ± SD (*n* = 6). *, *p* < 0.05 vs. zero.

^41^K infusion at the rate of 0.5 mg/h did not significantly alter plasma [K^+^] (Figure 1A). In contrast, the ^41^K infusion significantly increased the plasma ^41^K/^39^K ratio, expressed as a change from the baseline (∆^41^K/^39^K in ‰; Figure 1B). This increase was rapid during the first 10 min and slowed during the rest of the 60-min ^41^K infusion period, reaching ~1.83‰ at the end. Upon cessation of the ^41^K infusion, ∆^41^K/^39^K decreased rapidly during the first 10 min to ~0.85‰ and then slowly for the subsequent 4 h to ~0.53‰.

### 2.2. 3-C Modeling of Whole-Body K^+^ Distribution and Fluxes

**Figure 2:**
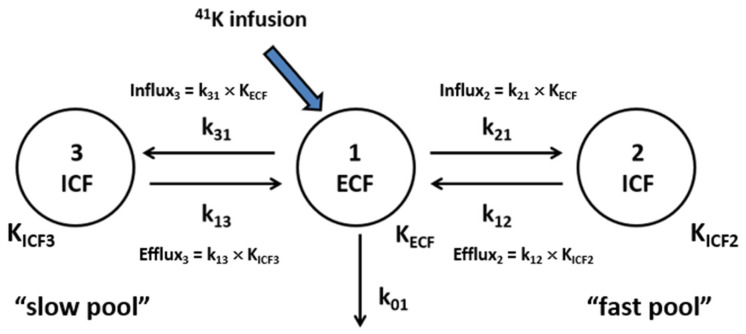
A 3-C model of K^+^ distribution and fluxes in vivo. K_ECF_ is the amount of K^+^ in the ECF; k_01_ is the rate constant for renal K^+^ excretion; k_21_ and k_12_ are the rate constants for K^+^ transport into and out of the “fast” ICF pool (ICF2), respectively; and k_31_ and k_13_ are the rate constants for K^+^ transport into and out of the “slow” ICF pool (ICF3), respectively.

The time course of the change in plasma Δ^41^K/^39^K was analyzed using a 3-C model of K^+^ distribution and fluxes (Figure 2), including the ECF (compartment 1) and two ICF (compartments 2 and 3) K^+^ pools, K^+^ fluxes between the pools, and renal K^+^ excretion. We assume a rapid equilibrium of the ECF with plasma, and plasma ∆^41^K/^39^K accurately reflects that in the ECF. The 3-C model fit the data quite well (Figure 1C), and the residuals (i.e., differences between the observed data and model prediction) were not statistically different from zero at all sampling time points except for 60 min (Figure 1D). In contrast, the 2-C model used in our previous publication [14] was not able to fit the data, resulting in significant deviations from the data at multiple time points (Supplemental Figure S1). The 3-C model parameters, identified in each animal, showed variations (i.e., fractional standard deviations [FSDs]) less than 61% for all parameters (Table 1, control). The size of the ECF K^+^ pool (i.e., K_ECF_) was estimated to be 0.49 ± 0.19 mEq/300 g BW, and the ICF2 and ICF3 K^+^ pools were estimated to be 3.7 ± 2.2 and 16.9 ± 1.6 mEq/300 g BW, respectively; the slow pool (i.e., K_ICF3_) was ~4.5 times larger than the fast pool (i.e., K_ICF2_), suggesting that the slow pool may represent the skeletal muscle, the major store of ICF K^+^ [1]. K_ICF3_ was very precisely identified with an FSD of 9.6%.

### 2.3. K^+^ Uptake by Individual Tissues

**Figure 3:**
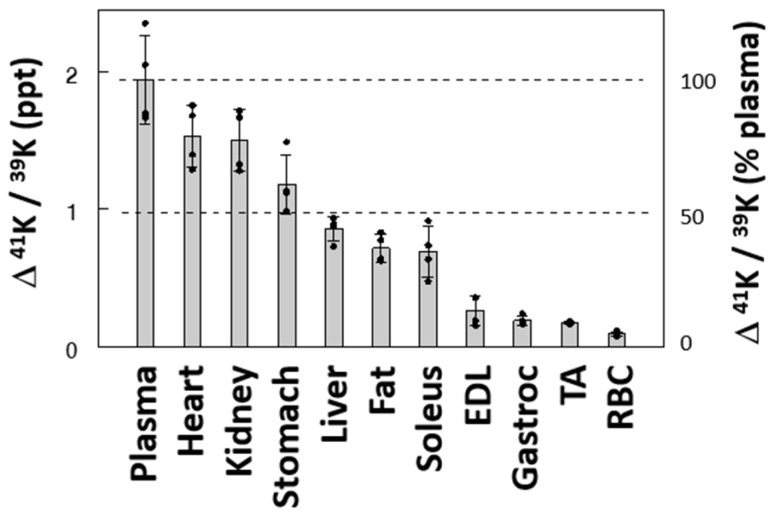
Effects of ^41^K infusion (0–60 min) on ∆^41^K/^39^K in plasma and individual tissues. Isotope ratios are expressed as increments (∆) from those before ^41^K infusion. Data are mean ± SD (*n* = 4). The bars and the error bars indicate means and SDs, respectively. EDL, extensor digitorum longus; TA, tibialis anterior; Gastroc, gastrocnemius muscle.

In a second series, ∆^41^K/^39^K was measured in individual tissues at the end of the 60 min ^41^K infusion (Figure 3). ∆^41^K/^39^K at 60 min varied substantially across tissues: 1.53, 1.50, and 1.18 ‰ in the heart, kidney, and stomach, respectively. These values were 79%, 77%, and 61% of the increase in plasma, respectively, suggesting these tissues rapidly take up K^+^ from the ECF. In contrast, skeletal muscles, such as extensor digitorum longus (EDL), tibialis anterior (TA), and gastrocnemius, and RBCs showed low ∆^41^K/^39^K, less than 14% of the plasma value, indicating these tissues are among those slowly exchanging K^+^ with the ECF under baseline equilibrium. ∆^41^K/^39^K in the liver and fat showed values between the two groups of tissues. Interestingly, ∆^41^K/^39^K in soleus muscle, mainly composed of type I fibers, was significantly larger than changes in muscles enriched in type II fibers (EDL, TA, and gastrocnemius), hinting at fiber-type-dependent K^+^ uptake, consistent with the higher sodium pump activity in type I vs. type II fibers [17]. Thus, these data demonstrate wide variations across tissues in terms of K^+^ uptake (or K^+^ exchange), classifying these tissues to “fast” vs. “slow” pools of the exchangeable ICF K^+^.

### 2.4. Effects of the Duration of K^+^ Infusion on Model Identification

**Figure 4:**
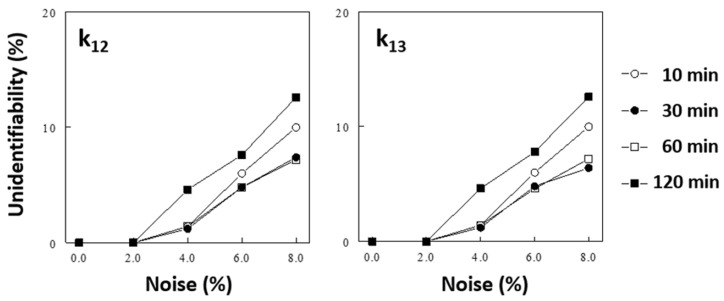
Effects of the duration of ^41^K infusion (and sampling schedule) on the identification of 3-C model parameters (i.e., k_12_ and k_l3_, see Supplemental Table S1 for effects on other parameters), assessed by a Monte Carlo simulation (see the Methods section). In this simulation, k_01_ (renal K^+^ excretion) was fixed and all parameters of the 3-C model were estimated from noise-added simulated data. The duration of ^41^K infusion simulated were 10, 30, 60, and 120 min (Protocol # 1, 2, 4, and 6, respectively; Supplemental Table S1), and the sampling schedules were adjusted. “Unidentifiability” was arbitrarily defined as the probability of estimated parameters being >3 times different from the true values (see the Methods section).

Our previous study [14] showed that increasing both the duration of the observation period and the number of samples taken improve the identification of the 3-C model parameters. We performed additional Monte Carlo simulations to test if the duration of ^41^K infusion impacts the identification of the 3-C model parameters. “Unidentifiability,” arbitrarily defined as the probability of identified parameters being more than 3 times different from the original values (see the Methods section), increased significantly as the noise levels increased. The duration of ^41^K infusion significantly impacted the unidentifiability of the model parameters (see Figure 4 for impacts on k_12_ and k_13_; also see Supplemental Table S1 for data on all parameters); a 30-min or 60-min infusion resulted in better identification of the model parameters than a shorter (10 min) or longer (120 min) infusion.

### 2.5. Effects of K^+^ Restriction on K^+^ Distribution and Fluxes

**Figure 5:**
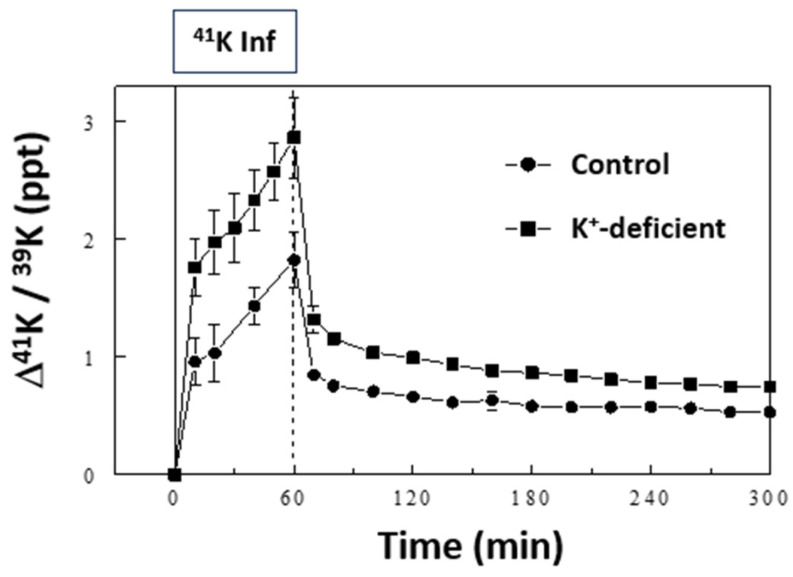
Effects of ^41^K infusion on plasma ∆^41^K/^39^K in K^+^-deficient rats compared with those in control rats. The data for control rats are the same as those in Figure 1B. Data are means ± SD (*n* = 6 for control and 7 for K^+^-deficient rats).

**Figure 6:**
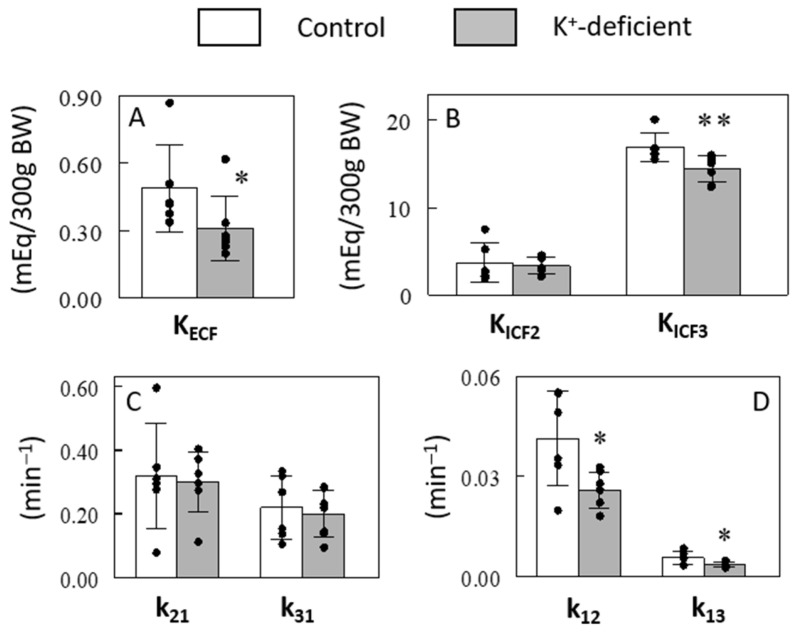
Effects of a 10-day K^+^ restriction on 3-C model estimated sizes of ECF (**A**) and ICF (**B**) K^+^ pools and rate constants for K^+^ fluxes into and out of the fast (**C**) and slow (**D**) ICF K^+^ pools. Data are means ± SD (*n* = 6 for control and 7 for K^+^-deficient rats). The bars and the error bars indicate means and SDs, respectively. *, *p* < 0.05; **, *p* < 0.01 vs. control.

We tested the ability of our model to identify changes in K^+^ pools and the kinetics of K^+^ transport associated with dietary K^+^ restriction by maintaining rats on a K^+^-free diet for 10 days prior to sampling. Based on the simulation results, a slight modification was made in the sampling schedule to include two additional samplings at 30 and 50 min (Protocol #4 vs. #3, Supplemental Table S1). K^+^ restriction resulted in a drop in plasma [K^+^] from 3.9 ± 0.3 mEq/L in the control-fed rats to 3.2 ± 0.5 mEq/L in the K^+^-deficient diet-fed rats (*p* < 0.05), as previously reported [14]. ^41^K infusion in these rats at the same rate of 0.5 mg/h resulted in higher plasma ∆^41^K/^39^K profiles during and after the ^41^K infusion compared to those in the control rats, although the overall pattern of changes was similar (Figure 5). Similar to the ^41^K infused control experiments, the 3-C model fit the K^+^-restricted experiment well, and the model parameters were robustly identified (Table 1). The ECF K^+^ pool (i.e., K_ECF_) was estimated to be lower by 37% in the K^+^-restricted rats compared to the control rats (*p* < 0.05; Figure 6A), consistent with the 16% lower plasma [K^+^] in these animals (*p* < 0.05). In addition, the size of ICF K^+^ pool decreased significantly in the slow pool (K_ICF3_; 15%, *p* < 0.01) but not in the fast pool (K_ICF2_; *p* > 0.05; Figure 6B). These modeling results are consistent with the findings that skeletal muscles are among the slow ICF K^+^ pools (Figure 3) and K^+^ restriction selectively decreases [K^+^] in skeletal muscles but not in other tissues [18,19]. Thus, the major changes in ICF K^+^ pool sizes detected by the 3-C model are validated by direct measurements. In addition, the 3-C analysis showed the rate constants for K^+^ effluxes (i.e., k_12_ and k_13_; *p* < 0.05, Figure 6D) but not for influxes (i.e., k_21_ and k_31_; *p* > 0.05, Figure 6C) significantly decreased after the 10-day K^+^ restriction in both the fast and slow ICF K^+^ pools. This novel finding was made possible by employing this methodological advance and sets the groundwork for analyzing the regulation of K^+^ efflux pathways separate from K^+^ influx pathways in vivo across tissues.

## 3. Discussion

We previously introduced a method for quantifying K^+^ distribution and fluxes in vivo using stable K^+^ isotopes [14]. The present study extends this new method to identify a more complex (i.e., 3-C vs. the previous 2-C) model of K^+^ distribution and fluxes in vivo, comprising multiple ICF K^+^ pools. This was made possible by increasing the observation period and number of samplings during the experiment based on previous computer simulations [14]. Our extended protocol allowed the 3-C model parameters to be robustly identified, detecting significant effects of a 10-day K^+^ restriction on the ICF K^+^ pool sizes and K^+^ fluxes (see below). In addition, we further extended the stable isotope approach to assess K^+^ uptake by individual tissues in vivo. We found wide variations in K^+^ uptake across tissues, classifying these tissues into “fast” vs. “slow” pools of ICF K^+^. This new method for assessing individual-tissue K^+^ uptake will allow one to study individual-tissue responses to physiological stimuli and relate tissue K^+^ transport activity to Na-K-ATPase content or other molecular events.

The variations in K^+^ uptake across tissues justify the expansion of the previous 2-C model, assuming a single homogenous ICF pool, to the current 3-C model comprising multiple ICF K^+^ pools. The 3-C model represents not only tissues in fast equilibrium with the ECF (“fast pool”) but also those in slow equilibrium with the ECF (“slow pool”) (Figure 2). In our previous study [14], the 2-C model underestimated the ICF K^+^ pool’s size; the model-estimated size of the ICF K^+^ pool (8.8 mEq) was only a half of the expected 16.8 mEq K^+^ pool for 300 g rats (=0.3 kg BW × 0.4 L/kg BW [ICF volume] × 140 mEq/L [ICF K^+^ concentration]). One possibility is that the 2-C analysis assuming a single ICF pool may detect fast ICF K^+^ pools but not slow pools [14]. To support this idea, in the present study, the 3-C analysis estimated the size of ICF pools to be 20.6 mEq (=K_ICF2_ + K_ICF3_), substantially larger than that estimated by the 2-C analysis and closer to the expected ICF pool size (or identical to the ICF pool size estimated assuming a 49% ICF volume). Thus, the 3-C model assuming two heterogenous ICF pools appears to accurately estimate the size of the ICF K^+^ pool. Similar 3-C models have been widely used in whole-body kinetic studies of many substrates and hormones [20,21]. However, to our knowledge, the present study is the first to explore 3-C modeling for K^+^ distribution and kinetics in vivo.

The 3-C model represents two heterogenous ICF K^+^ pools. Even the 3-C model is a simplified model, considering the wide variations in tissue K^+^ transport activities; any model is a simplified representation of a very complex real system. Nonetheless, the 3-C model describes different dynamics of heterogenous ICF K^+^ pools and produced quite interesting data regarding the effects of K^+^ restriction on K^+^ distribution and fluxes (see below). In addition, 3-C modeling indicates that the size of the slow ICF K^+^ pool is much larger than the fast K^+^ pool (Table 1 and Figure 6B), suggesting the slow pool may represent skeletal muscles, the major ICF K^+^ stores of the body [1]. Indeed, our estimates of individual-tissue K^+^ uptake revealed that skeletal muscles, such as EDL, TA, and gastrocnemius, are among the tissues that slowly take up K^+^ from plasma. Thus, two independent measurements (i.e., whole-body compartmental analysis and individual-tissue K^+^ fluxes) indicate that skeletal muscles serve as slow K^+^ pools, consistent with low blood flow in the skeletal muscle in the resting state in which the present study was performed. This situation may be changed during exercise, as muscle contractions increase local [K^+^] in the surrounding ECF, which stimulates Na-K-ATPase to clear the accumulated K^+^ from the surrounding ECF into the ICF facilitated by catecholamines [8]. Even though skeletal muscles slowly exchange K^+^ with the ECF in the resting state, its contribution as extrarenal tissues to K^+^ homeostasis is enormous because of its large mass and responses to hormones, such as insulin and epinephrine. The 3-C model precisely estimated the size of the slow pool, hypothetically representing skeletal muscles as discussed above, with fractional standard deviations of ~10%. Because the skeletal muscle is the major site of ECF K^+^ regulation, our stable isotope approach with 3-C modeling would be a powerful tool for K^+^ homeostatic studies by detecting changes in the slow ICF K^+^ pool as skeletal muscle K^+^ pools under various conditions (see below).

Our analysis with the 3-C modeling detected specific changes in K^+^ distribution and transport activities with a 10-day total K^+^ restriction. First, the K^+^ restriction decreased the size of the ECF K^+^ pool, consistent with a significant decrease in plasma concentration of K^+^ to compensate for the persistent renal loss of K^+^. In addition, the 10-day K^+^ restriction decreased the ICF K^+^ pool size, but only in the slow pool, representing skeletal muscles, not in the fast pool. Consistent with this finding, previous studies demonstrated that tissue [K^+^] decreased in skeletal muscles but remained unchanged in other (i.e., non-skeletal muscle) tissues during K^+^ restriction [18,19]. These data suggest a unique role of skeletal muscles to donate K^+^ to ECF when ECF [K^+^] falls. Furthermore, the modeling analysis showed significant effects of K^+^ restriction to decrease the rate constants for K^+^ fluxes out of the ICF pools with K^+^ restriction (Figure 6D). This effect was observed in both the fast and slow ICF K^+^ pools. This finding suggests a novel regulatory step, i.e., regulation of K^+^ efflux routes. We speculate that in the face of lower ECF [K^+^], depressed efflux would blunt the fall of ICF [K^+^] in response to the greater transmembrane potassium gradient. It is logical to assume that K^+^ effluxes are tightly controlled, considering that the ICF K^+^ pool is huge, 50 times larger than the ECF pool [1], and that a small shift of ICF K^+^ into the ECF could substantially increase ECF [K^+^]. In contrast, the rate constants for K^+^ transport into the ICF were not altered by K^+^ restriction (Figure 6C). This finding contrasts the research demonstrating that dietary K^+^ restriction decreases K^+^ fluxes into select muscle ICF pools via decreasing Na-K-ATPase abundance and activity resulting in a K^+^ shift from select muscle ICF pools to the ECF [1,19]. Decreased Na-K-ATPase activity in skeletal muscle was anticipated to decrease the rate constant for K^+^ flux into the slow ICF K^+^ pool, but we did not see such changes. It is likely that our modeling analysis could not detect changes in K^+^ transport activity into the ICF during K^+^ deficiency because the continuous K^+^ uptake via Na-K-ATPase across every cell of the body is very high compared to the depressed K^+^ uptake restricted to a subset of skeletal muscles. Future studies are warranted to directly examine the effects of K^+^ deficiency on K^+^ influx vs. K^+^ efflux in skeletal muscles.

We estimated K^+^ uptake by individual tissues by measuring Δ^41^K/^39^K in tissues at the end of the 60 min ^41^K infusion. These measurements of tissue Δ^41^K/^39^K may reflect K^+^ transport activities into the ICF in some but not all tissues because tissue samples for Δ^41^K/^39^K were collected 60 min after the start of ^41^K administration, which may be sufficient time for ^41^K transported into tissues to return to the ECF, especially in tissues with fast equilibrium with the ECF. In this case, ^41^K accumulation in tissues may reflect net ^41^K uptake, determined not only by K^+^ influx but also by K^+^ efflux activities. In skeletal muscles, which are in slow equilibrium with the ECF in resting states, ^41^K accumulation at 60 min stayed low, and we postulate that K^+^ efflux activity may not significantly affect ^41^K uptake and that ^41^K accumulation in tissues may be assumed to represent K^+^ influx activity. Future studies are warranted to determine the time course of increases (during ^41^K infusion) and decreases (following ^41^K infusion) in the ^41^K/^39^K ratios in individual tissues. The dynamics of Δ^41^K/^39^K in individual tissues may allow separate estimation of the rate constants for influx and efflux in each tissue, as demonstrated in red blood cells in our previous study [14]. If successful, such an approach would provide a useful tool for investigating how the rate constants for each tissue relate to the transporters involved (i.e., Na-K-ATPase vs. cotransporters vs. channels).

Quantitative estimates of the rate constants provide important insights into the roles of renal vs. extrarenal tissues in [K^+^] homeostasis. The rate constants for K^+^ fluxes into the ICF pools (0.32 for the fast pool and 0.22 for the slow pool) were 50–100 times larger than that for renal excretion (0.004) in the control group. This indicates very rapid exchange of K^+^ between the ECF and ICF, compared to renal excretion. Select extrarenal tissues altruistically rapidly take up K^+^ when ECF [K^+^] rises and release K^+^ when ECF [K^+^] falls. In contrast, whether in the control or K^+^ deficient states, the kidney carefully “fine tunes” K^+^ excretion to match K^+^ intake, which involve very small fluxes, rather than participating in the shift of K^+^ between ECF and ICF. Thus, these data support the concept that extrarenal tissues are primarily responsible for the acute minute-by-minute regulation of ECF [K^+^], especially during K^+^ absorption after a meal, while kidneys are responsible for maintaining total body K^+^ content in response to fluctuations in intake or extrarenal losses.

## 4. Materials and Methods

### 4.1. Animals and Catheterization

Male Wistar rats (280–300 g; ~9 weeks old) were purchased from Envigo and housed under controlled temperature (22 ± 2 °C) and lighting (12 h light, 6 AM–6 PM; 12 h dark, 6 PM–6 AM). The rats were maintained on a gel diet, containing 1% (control) or 0% K^+^ for 10 days prior to the experiment. The gel diets were prepared from a K^+^-deficient powdered diet (TD.88239; Envigo), as in our previous study [22]. The rats were housed individually in cages with wire floors and placed in tail restraints for 3 or 4 days prior to the experiment, required to protect the tail catheters during the experiments [9]. The animals were allowed unrestricted access to food and tap water provided in the vivarium to all the animals (our tap water contains ~5 mg/L of K^+^, estimated to be ~0.1% of dietary K^+^ intake). A tail-vein catheter for intravenous infusion and a tail-artery catheter for blood sampling were placed in the morning of the experiment (~7 AM) [9]. All procedures involving animals were approved by the Institutional Animal Care and Use Committee at the University of Southern California (Protocol # 21364).

### 4.2. ^41^K Infusion

The rats were studied in a conscious state after a 6-h fast. After taking basal blood samples through the tail-artery catheter at ~1 PM, ^41^K (ISOFLEX USA, San Francisco, CA, USA) was infused as in our previous study [14]; ^41^K (as KCl) was dissolved in normal saline (0.5 mg/mL) and infused for 1 h through the tail-vein catheter at the rate of 0.5 mg/h. Blood samples were collected before (basal) and at various times during and after the ^41^K infusion, as indicated elsewhere. Blood samples were rapidly spun for 1 min, and plasma samples were isolated and analyzed for the ^41^K/^39^K ratio. In addition, urine passed was collected from the bottom of cages, as previously described [9], to measure urinary K^+^ excretion. All samples were frozen and stored at −20 °C until analysis for the ^41^K/^39^K. In addition, total [K^+^] in plasma and urine samples were determined by flame photometry, as previously reported [9].

### 4.3. Ion Chromatography and Isotope Ratio Mass-Spectrometry for ^41^K/^39^K Determination

K^+^ was purified from plasma and urine samples for isotopic analyses using an automated high-pressure ion chromatography (IC) system, as previously described [14,23]. The accuracy of our chromatographic methods was verified by purifying and analyzing external standards (NIST SRM3141a [^41^K/^39^K ratio = ~0.0722] and SRM70b [^41^K/^39^K ratio = ~0.0721]) alongside unknown samples. Purified aliquots of K^+^ were analyzed in 2% HNO_3_ for their isotopic compositions on a Thermo Scientific Neptune Plus multi-collector inductively coupled plasma mass spectrometer (MC-ICP-MS) at Princeton University, using previously published methods [23,24,25]. The external reproducibility of our protocols (chromatography and mass spectrometry) as determined through replicate measurements of international standards is ~0.015%. Measured δ^41/39^K values were converted to ^41^K/^39^K ratios for numerical modeling using the equation for delta notation: δ^41/39^K = (^41^K/^39^K)_sample_/(^41^K/^39^K)_standard_−1, where we assume that the ^41^K/^39^K of the standard is equal to the natural abundance of K^+^ in nature (~0.0722).

### 4.4. Compartmental Analysis

Changes in the ^41^K/^39^K ratio from the baseline ratios (Δ^41^K/^39^K) were analyzed using a 3-C model that comprises the ECF and two ICF K^+^ pools, K^+^ fluxes between the ECF and ICF compartments, and renal K^+^ excretion (Figure 2). The 3-C model is represented by the following differential equations:

dy1(t)/dt = −(k_01_ + k_21_ + k_31_) × y1(t) + k_12_ × y2(t) × K_ICF2_/K_ECF_ + k_13_ × y3(t) × K_ICF3_/K_ECF_ + Inf(t)/K_ECF_ (1)

dy2(t)/dt = −k_12_ × y2(t) + k_21_ × y1(t) × K_ECF_/K_ICF2_ (2)

dy3(t)/dt = −k_13_ × y3(t) + k_31_ × y1(t) × K_ECF_/K_ICF3_ (3)

where y1(t) represents Δ^41^K/^39^K in the ECF and y2(t) and y3(t) represent Δ^41^K/^39^K in the two ICFs (i.e., ICF2 and ICF3, respectively); K_ECF_, K_ICF2_, and K_ICF3_ represent the amounts of K^+^ in the ECF and two ICF compartments, respectively; k_01_ is the rate constant for renal K^+^ excretion; k_21_ and k_12_ are the rate constants for K^+^ transport into and out of the ICF2 (“fast pool”), respectively; and k_31_ and k_13_ are the rate constants for K^+^ transport into and out of the ICF3 (“slow pool”), respectively. Inf(t) is equal to the ^41^K infusion rate from 0 to 60 min and becomes zero after 60 min. The model parameters (i.e., K_ECF_, K_ICF2_, K_ICF3_, k_01_, k_21_, and k_31_) were identified in individual animals from plasma Δ^41^K/^39^K profiles using a Levenberg–Marquardt nonlinear algorithm on MATLAB (R2019a Update 9). Although all six parameters could be identified from the data, we noted significant variations in estimated parameter values. To reduce the variability of estimated parameter values, urinary K^+^ excretion was directly measured in each rat by determining the volume of urine passed and its K^+^ concentration, and k_01_ was constrained in parameter identification using the relationship k_01_ = urinary K^+^ excretion/K_ECF_. The rest of the model parameters (K_ECF_, K_ICF2_, K_ICF3_, k_21_, and k_31_) could then be estimated from plasma Δ^41^K/^39^K profiles with acceptable variations. k_12_ was then calculated to be K_ECF_ × k_21_/K_ICF2_, and k_13_ to be K_ECF_ × k_31_/K_ICF3_. These equations are derived from Equations (2) and (3), respectively. For example, at steady state, dy2(t)/dt = 0 and y1(t) = y2(t), resulting in the relationship k_12_ = K_ECF_ × k_21_/K_ICF2_. Thus, k_12_ and k_13_, like other parameters, were estimated in each animal.

### 4.5. Monte Carlo Simulation

Our previous study showed that computer simulation is an effective way to optimize protocols, which saves time and animal experimentation [14]. After identifying the 3-C model in initial experiments using empirically determined protocols, we performed Monte Carlo simulations in MATLAB to test if the protocols can be modified in terms of the duration of ^41^K infusion and sampling times to improve the robustness of parameter identification. For this, a computer simulation was performed, basically as described in our previous study [14]. Briefly, the plasma Δ^41^K/^39^K profile was simulated using the 3-C model (and its estimated parameter values) under given protocols (i.e., duration of ^41^K infusion and sampling times). The simulated data were then added with random noises at different levels and subsequently analyzed to identify model parameters. A computer program repeated this process 1000 times at each noise level to estimate variations in the model parameters identified from noise-added simulated data. In this simulation study, we arbitrarily defined “unidentifiability” as the probability of estimated parameters being more than 3 times different from the true values, as in our previous study [14].

### 4.6. Statistical Analysis

All data are expressed as means ± SD. The significance of differences in the mean value was assessed by Students’ t-tests. *p* values were adjusted for multiple comparisons using the Bonferroni method. A *p* value less than 0.05 was considered statistically significant.

## 5. Conclusions

A 3-C model of K^+^ distributions and fluxes, comprising multiple (i.e., fast and slow) ICF K^+^ pools, was robustly identified based on experimental data with stable K^+^ isotopes. We show evidence that its slow pool represents the skeletal muscle, the major K^+^ stores. The 3-C model detected significant effects of K^+^ restriction on the ICF K^+^ pool sizes and K^+^ fluxes. In addition, we extended our stable isotope approach to assess K^+^ uptake by individual tissues in vivo, demonstrating wide variations in K^+^ uptake across tissues. Thus, the present study introduces new stable isotope approaches of quantifying whole-body and individual-tissue K^+^ fluxes, which can be used to identify mechanisms underlying the (patho)physiological regulation of K^+^ homeostasis.

## Figures and Tables

**Table 1:** 3-C model parameters identified by fitting the model to the plasma profile of Δ^41^K/^39^K in rats maintained on a control (1% K^+^) or K^+^-deficient (0% K^+^) diet.

	Control	K^+^-Deficient Diet	% Change
k_01_	0.0043 ± 0.0024	0.0003 ± 0.0003 ***	↓95%
k_21_	0.317 ± 0.166	0.298 ± 0.094	↓6%
k_12_	0.041 ± 0.014	0.026 ± 0.005 *	↓37%
k_31_	0.220 ± 0.100	0.199 ± 0.075	↓9%
k_13_	0.0058 ± 0.0020	0.0038 ± 0.0007 *	↓34%
K_ECF_	0.49 ± 0.19	0.31 ± 0.14 *	↓37%
K_ICF2 (fast)_	3.70 ± 2.22	3.33 ± 0.98	↓10%
K_ICF3 (slow)_	16.9 ± 1.6	14.4 ± 1.5 **	↓15%

k_01_ was estimated from renal K^+^ excretion measured from urine collected from the bottom of the cage (see the Methods section); five parameters (K_ECF_, K_ICF2_, K_ICF3_, k_21_, and k_31_) were identified by fitting the model to the data; and k_12_ and k_13_ were calculated as K_ECF_ × (k_21_/K_ICF2_) and K_ECF_ × (k_31_/K_ICF3_), respectively. Units are min^−1^ for k_01_, k_21_, k_12_, k_31_, and k_13_ and mEq/300g BW for K_ECF_, K_ICF2_, and K_ICF3_. Data are mean ± SD (*n* = 6 for control and 7 for K^+^ deficient diet). *, *p* < 0.05; **, *p* < 0.01; ***, *p* < 0.001 vs. control (two-tailed t-test for the rate constants and one-tailed t-test for K_ECF_, K_ICF2_, and K_ICF3_; *p* values were not corrected for multiple comparisons to increase the power of detecting changes, at the risk of false positives). ↓ represents a decrease.

## Data Availability

Raw data and MATLAB code will be provided upon request.

## Supplementary Materials

The following supporting information can be downloaded at https://www.mdpi.com/article/10.3390/ijms25179664/s1.
