# Supplementary material for: Kinetic Modeling of In Vivo K+ Distribution and Fluxes with Stable K+ Isotopes: Effects of Dietary K+ Restriction
Source: Int J Mol Sci. 2024 Sep 6;25(17):9664. doi: 10.3390/ijms25179664 (PMC11395305; doi:10.3390/ijms25179664)
Supplement: Supplementary file 1 [file ijms-25-09664-s001.zip › Supplemental Figure S1.pdf]

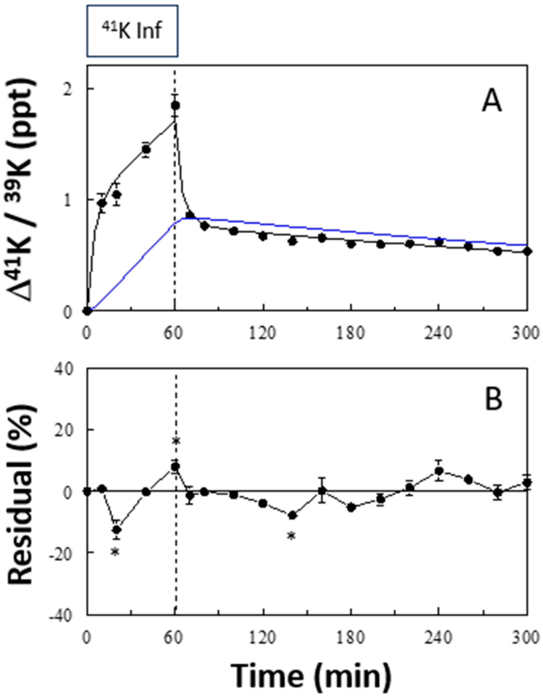

Supplemental Figure S1. 2-C model fit to the plasma profile of $\Delta^{41}K/^{39}K$ (**A**), shown together with the predicted profiles of $\Delta^{41}K/^{39}K$ in the ICF pool (blue curve), and residuals of the model fit (differences between observed and model-estimated values), expressed as % of observed values (**B**). Boxes indicate the ^{41}K infusion period. Isotope ratios are expressed as increments (Δ) from basal values. Data are means ± SEM (n = 6). *, $p < 0.05$ vs. zero (2-tailed t test; p values were adjusted for multiple comparisons by the Benjamini–Hochberg method).
